# SwissADME: a free web tool to evaluate pharmacokinetics, drug-likeness and medicinal chemistry friendliness of small molecules

**DOI:** 10.1038/srep42717

**Published:** 2017-03-03

**Authors:** Antoine Daina, Olivier Michielin, Vincent Zoete

**Affiliations:** 1 SIB Swiss Institute of Bioinformatics, Molecular Modeling Group, Quartier Sorge, Bâtiment Génopode, CH-1015 Lausanne, Switzerland; 2 Department of Oncology, Centre Hospitalier Universitaire Vaudois, CH-1015 Lausanne, Switzerland; 3 Ludwig Institute for Cancer Research, University of Lausanne, CH-1015 Lausanne, Switzerland

## Abstract

To be effective as a drug, a potent molecule must reach its target in the body in sufficient concentration, and stay there in a bioactive form long enough for the expected biologic events to occur. Drug development involves assessment of absorption, distribution, metabolism and excretion (ADME) increasingly earlier in the discovery process, at a stage when considered compounds are numerous but access to the physical samples is limited. In that context, computer models constitute valid alternatives to experiments. Here, we present the new SwissADME web tool that gives free access to a pool of fast yet robust predictive models for physicochemical properties, pharmacokinetics, drug-likeness and medicinal chemistry friendliness, among which in-house proficient methods such as the BOILED-Egg, iLOGP and Bioavailability Radar. Easy efficient input and interpretation are ensured thanks to a user-friendly interface through the login-free website http://www.swissadme.ch. Specialists, but also nonexpert in cheminformatics or computational chemistry can predict rapidly key parameters for a collection of molecules to support their drug discovery endeavours.

During the time- and resource-consuming processes of drug discovery and development, a large number of molecular structures are evaluated according to very diverse parameters in order to steer the selection of which chemicals to synthetize, test and promote, with the final goal to identify those with the best chance to become an effective medicine for the patients. The molecules must show high biological activity together with low toxicity. Equally important is the access to and concentration at the therapeutic target in the organism. The traditional way to consider pharmacokinetics (i.e. the fate of a therapeutic compound in the organism) is to break down the various effects that impact the access to the target into individual parameters. In turn, these ADME parameters (for Absorption, Distribution, Metabolism and Excretion) can be evaluated separately by dedicated methods. It has been demonstrated that early estimation of ADME in the discovery phase reduces drastically the fraction of pharmacokinetics-related failure in the clinical phases^1^. Computer models have been fostered as a valid alternative to experimental procedures for prediction of ADME, especially at initial steps, when investigated chemical structures are numerous but the availability of compounds is scarce^2^.

A large variety of *in silico* methods share the objective of predicting ADME parameters from molecular structure^3^. Noteworthy, the pioneer work of Lipinski *et al*. examined orally active compounds to define physicochemical ranges for high probability to be an oral drug (i.e. the drug-likeness)^4^. This so-called *Rule-of-five* delineated the relationship between pharmacokinetic and physicochemical parameters.

Whereas physicochemical parameters give a global description of the structure, molecules can be directly described by substructure searches. These techniques are at the root of Structural Alert^5^, the PAINS^6^ or the Lilly MedChem^7^ filters applied to cleanse chemical libraries from compounds most likely unstable, reactive, toxic, or prone to interfere with biological assays because unspecific frequent hitters, dyes or aggregators^8^.

Cheminformaticians developed different molecular descriptors mined from chemical structures. One of the most popular examples is the molecular fingerprint (FP), which consists in a sequence of bits defining the presence or absence of chemical features in a molecule. The FP2 method^9^ is one archetype of topological (or path-based) FP, which considers all fragments of the molecular structure following a linear path up to a given number of bonds. Every possible path is hashed to generate the bit string (i.e. the FP). A major advantage of FP is the efficiency by which computers handle such bit strings^10^, allowing for instance large-scale virtual screening or the rapid estimation of synthetic accessibility of molecules^11^. FP are also employed in classification models for ADME behaviours built by support vector machine (SVM) or Bayesian techniques^12^. Remarkably, computer-aided drug design (CADD) has been a pioneer field for the application of such machine learning technologies^13^.

Because most freely available *in silico* ADME tools focus on one specific property or model only, while generalist ADME packages are commercial software, we felt the need of gathering what we consider the most relevant computational methods to provide a global appraisal of the pharmacokinetics profile of small molecules. The methods were selected for robustness, speed, but also and importantly for ease of interpretation to enable efficient translation to medicinal chemistry through molecular design. Some of them were adapted with open-source algorithms to ensure freedom to operate for the global scientific community. Others are our own models developed and tested on purpose. When applicable, multiple predictions of the same parameter are provided to allow for a consensus view of a given property.

The SwissADME web tool presented here is freely accessible at http://www.swissadme.ch and meant for user-friendly submission and easy analysis of the results, also for nonexpert in CADD. Compared to the state-of-the art of free web-based tools for ADME and pharmacokinetics (e.g. pk-CSM^14^ and admetSAR^15^) and apart from unique access to proficient methods (e.g. iLOGP^16^ or the BOILED-Egg^17^), SwissADME strong points are, non-exhaustively: different input methods, computation for multiple molecules, and the possibility to display, save and share results per individual molecule or through global intuitive and interactive graphs. Finally, SwissADME is integrated in the SwissDrugDesign workspace. One-click interoperability gives access to various CADD tools developed by the Molecular Modeling Group of the SIB Swiss Institute of Bioinformatics, e.g. ligand-based virtual screening (SwissSimilarity^18^), biotarget prediction (SwissTargetPrediction^19^), molecular docking (SwissDock^20^), bioisosteric design (SwissBioisostere^21^), or molecular mechanics (SwissParam^22^).

## Submission Web page

**Figure 1:**
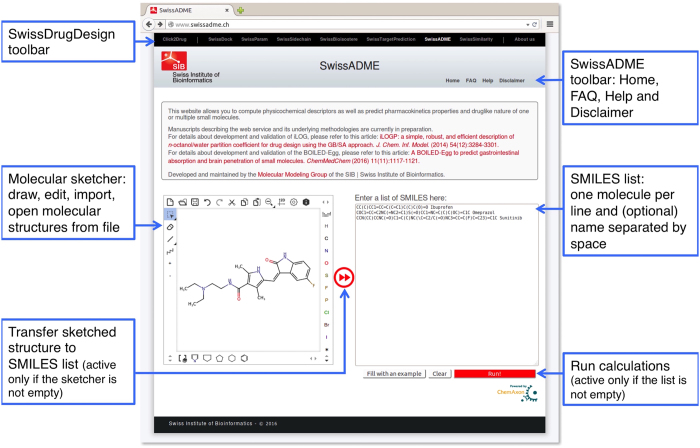
SwissADME submission page. The actual input is a list of SMILES, which contains one molecule per line with an optional name separated by a space. Molecules can be directly pasted or typed in SMILES format, or inserted through the molecular sketcher. The latter enables importing from databases, opening a local file or drawing a 2D chemical structure to be transferred to the list by clicking on the double-arrow button. When the list of molecules is ready to be submitted, the user can start the calculations by clicking on the “Run” button.

Accessing http://www.swissadme.ch in a web browser displays directly the submission page of SwissADME, where molecules to be estimated for ADME, physicochemistry, drug-likeness, pharmacokinetics and medicinal chemistry friendliness properties can be input. As shown in Fig. 1, a black toolbar at the top of the Web page allows the user to navigate within the different SwissDrugDesign tools. A second bar gives access to different information regarding SwissADME, among which the *FAQ* and *Help* pages as well as legal disclaimer and contacts.

The input zone itself comprises a molecular sketcher based on ChemAxon’s Marvin JS (http://www.chemaxon.com) that enables the user to import (from a file or an external database), draw, and edit a 2D chemical structure, and to transfer it to a list of molecules. This list, on the right-hand side of the submission page, is the actual input for computation. It can be edited as a standard text, allowing for typing or pasting SMILES. The list is made to contain one input molecule per line, defined by a SMILES and optionally a name separated by a space. If name is omitted, SwissADME will automatically provide an identifier.

Noteworthy, both buttons for transferring the sketch to SMILES list and for running the computation are dynamic, in the sense that they are active only if the action is possible. At the time of writing, one can expect a result in 1 to 5 seconds for a drug-like molecule.

Examples can be loaded in the SMILES list by clicking on the “Fill with an example” button.

## One-panel-per-molecule Output

**Figure 2:**
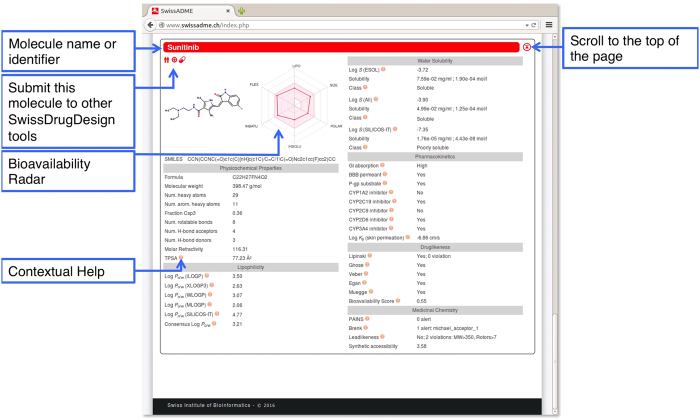
Computed parameter values are grouped in the different sections of the one-panel-par-molecule output (Physicochemical Properties, Lipophilicity, Pharmacokinetics, Drug-likeness and Medicinal Chemistry). The panel is headed by the molecule name and an up-arrow button to scroll to the top of the page. The molecule is first described by its chemical structure and canonical SMILES together with the Bioavailability Radar (see Fig. 3). Contextual help can be displayed by leaving the mouse over the radar or different question mark icons next to some parameters.

The output panels are loaded in the same Web page. There is one panel compiling all values for each molecule. It is filled immediately after calculation completion, one molecule after the other. This way it is possible to inspect the results for the first compounds without waiting for the whole list to be treated. This one-panel-per-molecule (Fig. 2) is headed by the molecule name and divided into different sections.

### Chemical Structure and Bioavailability Radar

**Figure 3:**
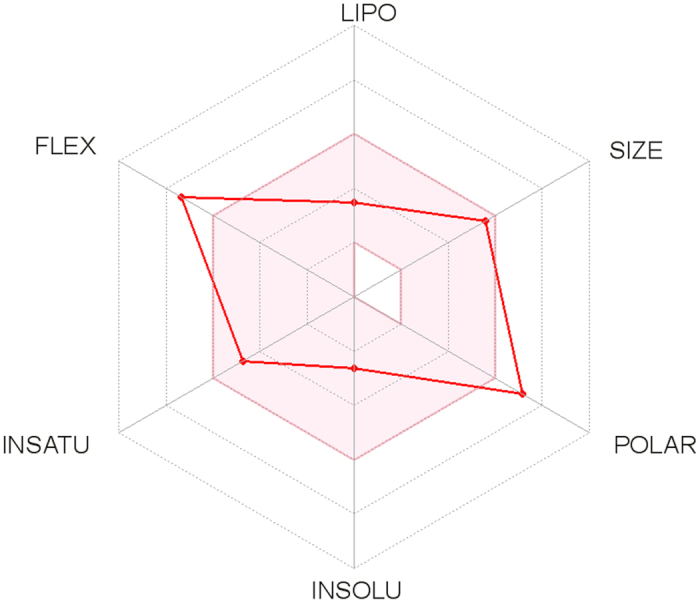
The Bioavailability Radar enables a first glance at the drug-likeness of a molecule. The pink area represents the optimal range for each properties (lipophilicity: XLOGP3 between −0.7 and +5.0, size: MW between 150 and 500 g/mol, polarity: TPSA between 20 and 130 Å^2^, solubility: log *S* not higher than 6, saturation: fraction of carbons in the sp^3^ hybridization not less than 0.25, and flexibility: no more than 9 rotatable bonds. In this example, the compound is predicted not orally bioavailable, because too flexible and too polar.

The first section, including two-dimensional chemical structure and canonical SMILES, is located below the title (Fig. 2). It shows on which chemical form the predictions were calculated (refer to *Computational Methods*). Moreover, our *Bioavailability Radar* is displayed for a rapid appraisal of drug-likeness (refer to Fig. 3). Six physicochemical properties are taken into account: lipophilicity, size, polarity, solubility, flexibility and saturation. A physicochemical range on each axis was defined by descriptors adapted from refs 23 and 24 and depicted as a pink area in which the radar plot of the molecule has to fall entirely to be considered drug-like. Leaving the mouse over the radar gives further information about the descriptors (see also *Physicochemical Properties and Computational Methods*).

### Physicochemical Properties

Simple molecular and physicochemical descriptors like molecular weight (MW), molecular refractivity (MR), count of specific atom types and polar surface area (PSA) are compiled in this section. The values are computed with OpenBabel^9^, version 2.3.0. The PSA is calculated using the fragmental technique called topological polar surface area (TPSA), considering sulfur and phosphorus as polar atoms^25^. This has proven a useful descriptor in many models and rules to quickly estimate some ADME properties, especially with regards to biological barrier crossing such as absorption and brain access^17^.

### Lipophilicity

The partition coefficient between *n*-octanol and water (log *P*_o/w_) is the classical descriptor for *Lipophilicity*. It has a dedicated section in SwissADME due to the critical importance of this physicochemical property for pharmacokinetics drug discovery^26^,^27^. Many computational methods for log *P*_o/w_ estimation were developed with diverse performance on various chemical sets. Common practice is to use multiple predictors either to select the most accurate methods for a given chemical series or to generate consensus estimation. The models behind the predictors should be as diverse as possible to increase the prediction accuracy through consensus log *P*_o/w_^28^. In that regard, SwissADME gives access to five freely available predictive models; i.e. XLOGP3, an atomistic method including corrective factors and knowledge-based library^29^; WLOGP, our own implementation of a purely atomistic method based on the fragmental system of Wildman and Crippen^30^; MLOGP, an archetype of topological method relying on a linear relationship with 13 molecular descriptors implemented from refs 31 and 32; SILICOS-IT, an hybrid method relying on 27 fragments and 7 topological descriptors (http://silicos-it.be.s3-website-eu-west-1.amazonaws.com/software/filter-it/1.0.2/filter-it.html, accessed June 2016); and finally iLOGP, our in-house physics-based method relying on free energies of solvation in *n*-octanol and water calculated by the Generalized-Born and solvent accessible surface area (GB/SA) model. iLOGP was benchmarked on two drug or drug-like external sets and performed equally as or better than six well-established predictors^16^. The consensus log *P*_o/w_ is the arithmetic mean of the values predicted by the five proposed methods.

### Water Solubility

Having a soluble molecule greatly facilitates many drug development activities, primarily the ease of handling and formulation^33^. Moreover, for discovery projects targeting oral administration, solubility is one major property influencing absorption^34^. As well, a drug meant for parenteral usage has to be highly soluble in water to deliver a sufficient quantity of active ingredient in the small volume of such pharmaceutical dosage^35^. Two topological methods to predict *Water Solubility* are included in SwissADME. The first one is an implementation of the ESOL model^36^ and the second one is adapted from Ali *et al*.^37^. Both differ from the seminal general solubility equation^38^ since they avoid the melting point parameter; the latter being challenging to predict. Moreover they demonstrate strong linear correlation between predicted and experimental values (R^2^ = 0.69 and 0.81, respectively). SwissADME third predictor for solubility was developed by SILICOS-IT. The linear correlation coefficient of this fragmental method corrected by molecular weight is R^2^ = 0.75 (http://silicos-it.be.s3-website-eu-west-1.amazonaws.com/software/filter-it/1.0.2/filter-it.html, accessed June 2016).

All predicted values are the decimal logarithm of the molar solubility in water (log *S*). SwissADME also provides solubility in mol/l and mg/ml along with qualitative solubility classes (please refer to *Computational Methods*).

### Pharmacokinetics

Specialized models, whose predictions are compiled in the *Pharmacokinetics* section, evaluate individual ADME behaviours of the molecule under investigation.

One model is a multiple linear regression, which aims at predicting the skin permeability coefficient (*K*_p_). It is adapted from Potts and Guy^39^, who found *K*_p_ linearly correlated with molecular size and lipophilicity (R^2^ = 0.67). The more negative the log *K*_*p*_ (with *K*_p_ in cm/s), the less skin permeant is the molecule.

The predictions for passive human gastrointestinal absorption (HIA) and blood-brain barrier (BBB) permeation both consist in the readout of the BOILED-Egg model^17^, an intuitive graphical classification model, which can be displayed in the SwissADME result page by clicking the red button appearing below the sketcher when all input molecules have been processed (refer to *Graphical Output*). Other binary classification models are included, which focus on the propensity for a given small molecule to be substrate or inhibitor of proteins governing important pharmacokinetic behaviours.

The knowledge about compounds being substrate or non-substrate of the permeability glycoprotein (P-gp, suggested the most important member among ATP-binding cassette transporters or ABC-transporters) is key to appraise active efflux through biological membranes, for instance from the gastrointestinal wall to the lumen or from the brain^40^. One major role of P-gp is to protect the central nervous system (CNS) from xenobiotics^41^. Importantly as well, P-gp is overexpressed in some tumour cells and leads to multidrug-resistant cancers^42^.

Also essential is the knowledge about interaction of molecules with cytochromes P450 (CYP). This superfamily of isoenzymes is a key player in drug elimination through metabolic biotransformation^43^. It has been suggested that CYP and P-gp can process small molecules synergistically to improve protection of tissues and organisms^44^. One can estimate that 50 to 90% (depending on the authors) of therapeutic molecules are substrate of five major isoforms (CYP1A2, CYP2C19, CYP2C9, CYP2D6, CYP3A4)^45^,^46^. Inhibition of these isoenzymes is certainly one major cause of pharmacokinetics-related drug-drug interactions^47^,^48^ leading to toxic or other unwanted adverse effects due to the lower clearance and accumulation of the drug or its metabolites^49^. Numerous inhibitors of the CYP isoforms have been identified. Some are affecting different CYP isoforms, while other compounds show selectivity for specific isoenzymes^50^. It is therefore of great importance for drug discovery to predict the propensity with which the molecule will cause significant drug interactions through inhibition of CYPs, and to determine which isoforms are affected.

SwissADME enables the estimation for a chemical to be substrate of P-gp or inhibitor of the most important CYP isoenzymes. We applied the support vector machine algorithm (SVM)^51^ on meticulously cleansed large datasets of known substrates/non-substrates or inhibitors/non-inhibitors (for details, see *Computational Methods*). In similar contexts, SVM was found to perform better than other machine-learning algorithms for binary classification^40^,^52^. The models return “Yes” or “No” if the molecule under investigation has higher probability to be substrate or non-substrate of P-gp (respectively inhibitor or non-inhibitor of a given CYP). The statistical performance of the classification models is given in Table 1, in comparison with previous SVM models on the same targets. We restricted the benchmark to state-of-the-art methods, published after 2010.

The quantification of the models performance is not straightforwardly comparable, because the training sets are different, most of the published models are less than 10-fold cross-validated and some statistical parameters are missing. Nevertheless, the SwissADME classifiers are competitive with previous models in term of robustness, with cross-validation accuracy (ACC_CV_) grossly at the same level. Furthermore, cross-validated areas under receiver operating characteristic (ROC) curves (AUC_CV_) are equal to the corresponding values found in the literature. Likewise, external prediction power is difficult to compare, as each test set includes different molecules. However, the predictive capacity of SwissADME classifiers is grossly equivalent to the related SVM methods, both in terms of external accuracy (ACC_ext_) and external area under ROC curve (AUC_ext_). The models for which external validation was not found (P-gp of refs 15 and 53) have to be taken with extreme caution since they possibly suffer from overfitting biases. Noteworthy, some of the published models (e.g. CYP2C9 and CYP3A4 of ref. 54 or CYP2C9 of ref. 55) were built on severely unbalanced training sets and tested on clearly unbalanced external sets. As demonstrated by Carbon-Mangels *et al*.^56^ the relevance of machine-learning classification methods, and especially SVM, are negatively impacted by datasets with one significantly more populated class. In that case, accuracy measurements are overestimated and prone to mislead the construction and the evaluation of the model. Moreover, some SVM models were published with small training and test sets (P-gp of refs 15 and 57), which imply questionable capacity of generalization and broadness of applicability domains. We emphasize that for SwissADME classifiers, both training and test sets were carefully cleansed and checked for size, diversity and balance between classes. Furthermore, our SVM models rely merely on molecular and physicochemical descriptors generated by SwissADME. We believe that this improves robustness and sustainability of the underlying methodologies. In particular, not using molecular fingerprints, molecular graphs or other structural descriptions can be an handicap to generate high statistical values but should also limit overfitting biases and yield more generalist predictive models, not necessarily influenced by specific chemical scaffolds or moieties. In our practice, these well-performing models able to estimate important ADME behaviours are of great support for pharmacokinetics optimization and evaluation of small molecules.

### Drug-likeness

As defined earlier, “drug-likeness” assesses qualitatively the chance for a molecule to become an oral drug with respect to bioavailability. Drug-likeness was established from structural or physicochemical inspections of development compounds advanced enough to be considered oral drug-candidates. This notion is routinely employed to perform filtering of chemical libraries to exclude molecules with properties most probably incompatible with an acceptable pharmacokinetics profile. This SwissADME section gives access to five different rule-based filters, with diverse ranges of properties inside of which the molecule is defined as drug-like. These filters often originate from analyses by major pharmaceutical companies aiming to improve the quality of their proprietary chemical collections. The Lipinski (Pfizer) filter is the pioneer rule-of-five implemented from ref. 4. The Ghose (Amgen), Veber (GSK), Egan (Pharmacia) and Muegge (Bayer) methods were adapted from refs 58, 59, 60, 61, respectively. Multiple estimations allow consensus views or selection of methods best fitting the end-user’s specific needs in terms of chemical space or project-related demands. Any violation of any rule described here appears explicitly in the output panel.

The Abbot Bioavailability Score^62^ is similar but seeks to predict the probability of a compound to have at least 10% oral bioavailability in rat or measurable Caco-2 permeability. This semi-quantitative rule-based score relying on total charge, TPSA, and violation to the Lipinski filter defines four classes of compounds with probabilities of 11%, 17%, 56% or 85%. Like the other methods in this section, it primary focuses on the fast screening of chemical libraries, to select the best molecules to be purchased, synthetized or promoted at a further stage of a medicinal chemistry project.

### Medicinal Chemistry

The purpose of this section is to support medicinal chemists in their daily drug discovery endeavours. Two complementary pattern recognition methods allow for identification of potentially problematic fragments. PAINS (for pan assay interference compounds, a.k.a. frequent hitters or promiscuous compounds) are molecules containing substructures showing potent response in assays irrespective of the protein target. Such fragments, yielding false positive biological output, have been identified by Baell *et al*.^6^ in analysing six orthogonal assays and breaking down the molecules active on 2 or more assays into 481 recurrent fragments, considered as potentially leading to promiscuous compounds. SwissADME returns warnings if such moieties are found in the molecule under evaluation.

Besides, we implemented Structural Alert, which consists in a list of 105 fragments identified by Brenk *et al*.^5^ to be putatively toxic, chemically reactive, metabolically unstable or to bear properties responsible for poor pharmacokinetics. In SwissADME, it is possible to have a chemical description of the problematic fragments found in a given molecule by flying over the “question mark” icon appearing after the fragment list. This is implemented for both PAINS and Brenk filters. By applying these and other physicochemical filters to design screening libraries, Brenk *et al*.^5^ observed that most of the remaining compounds satisfy criteria for “leadlikeness”. This concept is similar to drug-likeness, yet focusing on physicochemical boundaries defining a good lead, i.e. a molecular entity suitable for optimization. By definition, leads are subjected to chemical modifications that will most likely increase size and lipophilicity^63^. As a consequence, leads are required to be smaller and less hydrophobic than drug-like molecules. Since it is crucial for a chemist to judge whether a given molecule is suitable to initiate lead optimization, in addition to structural filters, we implemented a rule-based method for leadlikeness, which was adapted from ref. 64.

One of the key aspects of CADD activities is to help the selection of the most promising virtual molecules that will be synthetized and submitted to biological assays or other experiments. Synthetic accessibility (SA) is a major factor to consider in this selection process. Obviously, for a reasonable number of molecules, medicinal chemists are the best able to determine SA. However, when too many molecular structures prevent an expert evaluation, *in silico* estimation can be used for pre-filtering. Ertl & Schuffenhauer^11^ proposed a fingerprint-based approach for SA estimation but including closed-source information about fingerprint definition that prevents a straightforward implementation in our tool open to the scientific community. As a consequence, we have built our own fragmental method by analysing more than 13 millions compounds immediately deliverable by vendors. We assumed that the most frequent molecular fragments (technically, FP2 bits, refer to *Computational Methods*) in this large collection indicates a probably high SA, while rare fragments imply a difficult synthesis. For a given molecule, the fragmental contributions to SA are summed and corrected by the terms describing size and complexity, such as macrocycles, chiral centres, or spiro functions as defined by Ertl & Schuffenhauer^11^. After normalization, the SA Score ranges from 1 (very easy) to 10 (very difficult). To assess the performance of the method developed for SwissADME, we retrieved two test sets of SA previously published. Both sets involved external molecules, whose difficulty of synthesis was marked from 1 to 10 by nine^11^ and four^65^ medicinal chemists, respectively. The averaged expert score can then be compared to an *in silico* SA Score. As seen in Table 2, the predictive capacity of all three methods appears very dependent of the test set. Indeed the SAs of set 1, smaller and evaluated by more chemists, turned out to be much more robustly predictable than set 2. Human evaluation of synthetic complexity is undeniably subjective and relies on individual chemist’s training and experience. However, significant linear correlation and small errors — especially with SwissADME SA Score that outperformed the reference methods on both sets with smaller errors, and equal or higher linear correlation coefficients — demonstrate how this simple and fast methodology can help prioritizing molecules to synthetize.

## Graphical output

**Figure 4:**
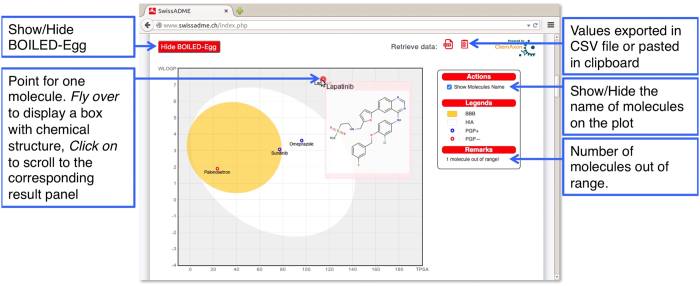
The BOILED-Egg 17 **allows for intuitive evaluation of passive gastrointestinal absorption (HIA) and brain penetration (BBB) in function of the position of the molecules in the WLOGP-*versus*-TPSA referential.** The white region is for high probability of passive absorption by the gastrointestinal tract, and the yellow region (yolk) is for high probability of brain penetration. Yolk and white areas are not mutually exclusive. In addition the points are coloured in blue if predicted as actively effluxed by P-gp (PGP+) and in red if predicted as non-substrate of P-gp (PGP−). For an interactive analysis, the user can leave the mouse over a dot to show the structure of the molecule and click on the dot to scroll to the corresponding output panel. In this example, Lapatinib is predicted as not absorbed and not brain penetrant (outside the Egg), Omeprazol is predicted well-absorbed but not accessing the brain (in the white) and PGP+ (blue dot), Sunitinib is predicted as passively crossing the BBB (in the yolk), but pumped-out from the brain (blue dot), and Palonosetron is predicted as brain-penetrant (in the yolk) and not subject to active efflux (red dot). One molecule is predicted not absorbed and not BBB permeant because outside of the range of the plot (streptomycin with a TPSA of 331.43 Å 2 and a WLOGP of −7.74).

After all calculations completed, the “Show BOILED-Egg” red button appears below the sketcher to display the graphical output on the same page (as exemplify in Fig. 4). This consists primarily in the BOILED-Egg, an intuitive method to predict simultaneously two key ADME parameters, i.e. the passive gastrointestinal absorption (HIA) and brain access (BBB). Although conceptually very simple as it relies on two physicochemical descriptors only (WLOGP and TPSA, for lipophilicity and apparent polarity), this classification model was built with extreme care regarding statistical significance and robustness^17^. As shown in Fig. 4, the egg-shaped classification plot includes the yolk (i.e. the physicochemical space for highly probable BBB permeation) and the white (i.e. the physicochemical space for highly probable HIA absorption). Both compartments are not mutually exclusive and the outside grey region stands for molecules with properties implying predicted low absorption and limited brain penetration. In practice, the BOILED-Egg has proven straightforward interpretation and efficient translation to molecular design in a variety of drug discovery settings. Whereas the predictive power of the BOILED-Egg is broad in term of chemical space, it is restricted to passive penetration through gastro-intestinal wall and BBB. We took benefit of its implementation within SwissADME to enrich the graphical output with the prediction of P-gp substrate, which is the most important active efflux mechanism involved in those biological barriers^40^ (refer to the SVM model described in *Pharmacokinetics*). As a result, the user conveniently obtains on the same graph a global evaluation about passive absorption (inside/outside the white), passive brain access (inside/outside the yolk) and active efflux from the CNS or to the gastrointestinal lumen by colour-coding: blue dots for P-gp substrates (PGP+) and red dots for P-gp non-substrate (PGP−).

Contrary to the one-panel-per-molecule concept for other parameters, the graphical output includes prediction for all molecules submitted to SwissADME, thus additional capacities were implemented to enable interactive navigation and easy evaluation. Flying over a specific point makes a semi-transparent box appear including the name and structure of the molecule. Clicking on a specific point makes the page scroll to the corresponding output panel including all predictions for the molecule. Getting back to the graphical output is achieved by clicking on the red up-arrow on the top-right corner of the panel. Moreover, on the right of the plot are displayed possible actions (at the moment, to show the name of the molecules on the graph, only), legends and possible remarks (the number of molecules outside the range of the plot). The user may want to hide the plot by clicking the “Hide BOILED-Egg” red button.

## Help, saving and interoperability

SwissADME is user-friendly and has been conceived for a variety of users, including nonexpert in cheminformatics or computational chemistry. Efforts were put to insure ease of input and interpretability of output. These aspects are supported by the *Help* page, which provides technical guidance on the usage of the website and by the *FAQ*, where we collected not only technical but also scientific and general questions. Contextual help about specific items can be displayed by leaving the mouse over question marks or some boxes on the pages.

Moreover, SwissADME is an integral part of the SwissDrugDesign program, an ambitious initiative driven by the Molecular Modeling Group of the SIB Swiss Institute of Bioinformatics that aims at providing a collection of free Web-based tools covering many aspects of CADD. As such, SwissADME users can access the different Web sites, through the black toolbar at the top of the page. By clicking on the name of the tool, a new tab opens with the corresponding submission page. We have undertaken the implementation of more advanced interoperability capabilities. At the time of writing this manuscript, three icons were placed top left of the output panel (below the name of the molecule, see Fig. 2). They allow one-click submission of the molecule to SwissTargetPrediction (for reverse screening to predict protein target, by clicking on the red target), SwissSimilarity (for ligand-based virtual screening, by clicking on the red twins) and to re-submit the molecule to SwissADME (for instance to get a single compound graphical output, by clicking the red pill). Reversely, other SwissDrugDesign websites include the SwissADME pill icon for estimation of ADME, pharmacokinetics, drug-likeness and medicinal chemistry friendliness regarding a small molecule output of any CADD process.

Importantly, the user has the opportunity to save or share SwissADME results in files (CSV) or by copying the values in the clipboard of the local computer and pasting in text or spreadsheet applications for further processing.

## Conclusions

The SwissADME Web tool enables the computation of key physicochemical, pharmacokinetic, drug-like and related parameters for one or multiple molecules. In one hand, efforts were put in the backend to embed free open-access and fast predictive models showing statistical significance, predictive power, intuitive interpretation, and straightforward translation to molecular design. These models are adapted from published renowned approaches or in-house original methods, specially developed and thoroughly benchmarked. On the other hand, we focused on an ergonomic and user-friendly graphical interface for the cost- and login-free Web site http://www.swissadme.ch. The latter enables easy input and efficient analysis of the output through interactive capabilities, and does not require any prior knowledge in CADD. Moreover, interoperability allows for direct access to other SwissDrugDesign web tools, including SwissSimilarity^66^ (virtual screening), SwissBioisostere^21^ (ligand-based design), SwissTargetPrediction^19^ (prediction of biotargets), SwissDock^20^ (molecular docking), SwissSideChain^67^ (protein modeling) and SwissParam^22^ (molecular mechanics).

As a result, SwissADME has been designed to support the entire community (specialists and nonexperts) in their drug discovery endeavours.

## Computational methods

### Programming and scripting

The SwissADME website was written in HTML, PHP5, and JavaScript, whereas the backend of computation was mainly coded in Python 2.7. The use of additional libraries or software for specific tasks is mentioned in the corresponding paragraph.

### Submission Page

The molecule inputted through the sketcher Marvin JS (version 16.4.18, 2016, www.chemaxon.com) are converted into SMILES by JChem Web Services (version 14.9.29, 2013, www.chemaxon.com) installed on one of our servers. This on-the-fly conversion allows seamless paste of SMILES in the input list. The user has the possibility to edit this list as a standard text, e.g. to modify SMILES or add a name to the molecule. Upon calculation submission by clicking the “Run” button, the SMILES of each molecule is canonicalised by OpenBabel (version 2.3.0, 2012, http://openbabel.org)^9^ and processed individually. Several actions are performed through JChem Web Services APIs. First hydrogen atoms are added to the molecular structure, which is dearomatised (i.e. kekulised), neutralised and checked by the Standardizer API. Then a tridimensional conformation is generated though the StringMolExport function with the Clean3D option and stored in MOL2 format. Besides, a two-dimensional image created through the MolConverter API is displayed on demand when scrolling the output web page.

### One-panel-per-molecule Output

The *Bioavailability Radar* in the first section of the One-panel-per-molecule output complements the two-dimensional image from the JChem webserver and the canonical SMILES calculated by OpenBabel. We use the JpGraph PHP library (version 3.5.0b1, 2016, http://jpgraph.net) to produce the radar plot, which bears six axes for six important properties for oral bioavailability. Each property is defined by a descriptor of SwissADME and a range of optimal values is depicted as a pink area. The latter is inspired from commonly accepted bioavailability and drug-likeness guidelines^23^,^24^. For saturation, the ratio of sp^3^ hybridized carbons over the total carbon count of the molecule (Fraction Csp3) should be at least 0.25. For size, the molecular weight (MW calculated by OpenBabel) should be between 150 and 500 g/mol. For polarity, the TPSA^25^ should be between 20 and 130Å^2^. For solubility, log *S* (calculated with the ESOL model^36^) should not exceed 6. For lipophilicity, XLOGP3^29^ should be in the range from −0.7 to +6.0. For flexibility, the molecule should not have more than 9 rotatable bonds. To be estimated as drug-like, the red line of the compound under study must be fully included in the pink area. Any deviation represents a suboptimal physicochemical property for oral bioavailability.

All descriptors and molecular parameters of the *Physicochemical Properties* section are computed through the OpenBabel API (version 2.3.0, 2012, http://openbabel.org)^9^. Noteworthy, the topological polar surface area (TPSA) is strictly based on the fragmental system provided by Ertl *et al*.^25^ including polar sulfur and phosphorus atoms.

Multiple freely available computational methods to predict *n*-octanol/water partition coefficient (log *P*_o/w_) values are made available in the *Lipophilicity* section. iLOGP (for implicit log *P*) is an in-house physics-based methods relying on Gibbs free energy of solvation calculated by GB/SA in water and *n*-octanol. Generalized-born (GB) parameters are computed through the GBMV2 method^68^ and solvent-accessible surface area (SA) is the analytical approximation generated by CHARMM (version c36b1, 2011, https://www.charmm.org)^69^. The iLOGP implemented in SwissADME corresponds to Model9 of the seminal publication^16^, which was trained on 11,993 molecules (r = 0.72, MAE = 0.89, and RMSE = 1.14 against experimental log *P*). 5-fold crossvalidation ensured robustness (q^2^_CV_ = 0.52, MAE_CV_ = 0.89, and RMSE_CV_ = 1.14) and external test benchmarks showed the excellent predictive power and extended applicability domain compared to well-established methods. XLOGP3 values are obtained through the command-line Linux program (version 3.2.2, courtesy of CCBG, Shanghai Institute of Organic Chemistry) including the knowledge-based corrections^29^. WLOGP is our own implementation of the atomistic method developed by Wildman and Crippen^30^. MLOGP values are computed through an in-house implementation of Moriguchi’s topological method^31^,^32^. SILICOS-IT is the log *P*_o/w_ estimation returned by executing the FILTER-IT program (version 1.0.2, 2013, http://silicos-it.be.s3-website-eu-west-1.amazonaws.com/software/filter-it/1.0.2/filter-it.html). Finally, SwissADME gives a consensus log *P*_o/w_ value, which is the arithmetic mean of the five predictive values mentioned above.

Similarly to lipophilicity, the *Water Solubility* section includes multiple predictive methods for the user to choose between the most accurate model for a given chemical series and an averaged consensus value. The ESOL model^36^ is a QSPR model establishing the linear relationship between log *S* and five molecular parameters, i.e. MW, the number of rotatable bonds, the fraction of aromatic heavy atoms and Daylight’s CLOGP. Because the lipophilicity descriptor is not freely available, the implementation of ESOL in SwissADME replaces CLOGP by XLOGP3 as parameter in the linear equation to predict log *S*. XLOGP3 is known to perform well on external datasets and to return similar predictions as CLOGP^28^. The other three parameters were computed with OpenBabel. Likewise, Ali *et al*.^37^ linked log *S* with log *P*_o/w_ and TPSA. The model implemented in SwissADME corresponds to the model 3 of the original publication, with XLOGP3 as lipophilicity descriptor. The third solubility method available in SwissADME is the log *S* estimated by the FILTER-IT program (version 1.0.2, 2013, http://silicos-it.be.s3-website-eu-west-1.amazonaws.com/software/filter-it/1.0.2/filter-it.html). This prediction is based on a system of 16 fragmental contributions modulated by the squared root of MW. All three models are predicting log *S* values, which are also translated within SwissADME into solubility in mol/l and mg/ml. Finally a qualitative estimation of the solubility class is given according to the following log *S* scale: insoluble <−10 <poorly <−6 <moderately <−4 <soluble <−2 <very <0 <highly.

The *Pharmacokinetics* section proposes one linear method for skin permeation, which relies on the simple QSPR model by Potts and Guy^39^ linking the decimal logarithm of the skin permeability coefficient (log *K*_*p*_ in cm/s) with MW and log *P*_o/w_. The model implemented in SwissADME uses XLOGP3 as lipophilicity descriptor. Besides, most of the models in this section are machine-learning binary classifiers for important ADME behaviours. Passive gastro-intestinal (HIA) absorption and blood-brain barrier (BBB) permeation are predicted with the BOILED-Egg model, which defines favourable and unfavourable zones in the log *P*_o/w_ *versus* PSA physicochemical space for passive diffusion through both physiological barriers^17^. The classification showed 10-fold cross-validation accuracy of 92% and 88% for BBB and HIA, respectively (refer to *Graphical Output*).

Six other classification models are part of the *Pharmacokinetics* section to predict the propensity of the molecule under investigation to be substrate or inhibitor of important pharmacokinetics-related proteins, for which large diverse and balanced datasets were retrieved and meticulously cleansed. For P-glycoprotein1 (P-gp), the training set consists of 521 substrates and 512 non-substrates extracted from the Metrabase database^70^ (http://www-metrabase.ch.cam.ac.uk, accessed January 2016), whereas the test set was obtained from ref. 71. To ensure truly external validation, molecules overlapping with the training set were removed from the test set, which finally includes 215 substrates and 200 non-substrates. For CYP major isoforms, all datasets were those of Veith *et al*.^50^ and downloaded from the PubChem database^72^ (http://pubchem.ncbi.nlm.nih.gov, accessed February 2016). In case of unbalanced dataset (all except CYP1A2 and CYP2C19), sufficient chemical diversity was guaranteed by clustering with the Ward method and a reciprocal nearest neighbour (RNN) algorithm^73^, the more populated class to lessen. The number of molecules (described by circular fingerprints) of the large class is reduced by defining clusters with the JKlustor program (version 14.9.29, 2014, http://www.chemaxon.com). Only the centre of each cluster (i.e. the molecule that has the smallest sum of dissimilarities to the other molecules in the cluster) is included in the training or test set to balance. As a result, the training sets involved respectively 4301 CYP1A2 inhibitors and 4844 CYP1A2 non-inhibitors; 4284 CYP2C19 inhibitors and 4988 CYP2C19 non-inhibitors; 2940 CYP2C9 inhibitors and 3000 CYP2C9 non-inhibitors; 1814 CYP2D6 inhibitors and 1850 CYP2D6 non-inhibitors; and 3758 CYP3A4 inhibitors and 3760 CYP3A4 non-inhibitors. The test sets involved respectively 1412 CYP1A2 inhibitors and 1588 CYP1A2 non-inhibitors; 1386 CYP2C19 inhibitors and 1614 CYP2C19 non-inhibitors; 1020 CYP2C9 inhibitors and 1055 CYP2C9 non-inhibitors; 528 CYP2D6 inhibitors and 540 CYP2D6 non-inhibitors; and 1289 CYP3A4 inhibitors and 1290 CYP3A4 non-inhibitors.

SwissADME’s backend calculations were ran to generate 50 molecular and physicochemical descriptors per molecule (described in the Supplementary Table S1). For a given model, a descriptor was rejected if non-zero values for all molecules in the training set are less than 20% or if the coefficient of variation is less than 3%. In case of correlation higher than 0.9 between remaining descriptors, a selection is made based on F-score. The selected descriptors for each model are shown in Supplementary Tables S2–S7. These tables also include the minimum and maximum values for each descriptor among all molecules used in the training. This enables beholding the broadness of physicochemical space involved and the applicability domain of the SVM models. The predictive capability of each model can be further appraised on Supplementary Table S8, where external accuracy was split in sensitivity and specificity to ensure that positive and negative molecules are predicted with the same level of robustness. The final training and test sets with selected descriptors were normalized and the respective model ready to be built. First, the libSVM support vector machine python library (version 3.20, 2015, https://www.csie.ntu.edu.tw/~cjlin/libsvm/)^74^ was used for multi-step grid-based optimization of the best coefficients for the above-selected descriptors as well as for the soft-margin permissivity (C) and the hyper-parameter (ϒ) of the RBF Gaussian kernel function. The 10-fold crossvalidated accuracy (ACC_CV_) for each model was so maximized and AUC_CV_ was calculated. In a second step, the so-built models were used on the external test sets (normalized according to the training set) in order to evaluate predictive power in terms of external accuracy (ACC_ext_) and AUC_ext_. All final SVM models were stored in separate files, which are read through the libSVM API upon SwissADME job submission.

#### The *Drug-likeness* section includes six rule-based methods

The Lipinski rule-of-five is exactly as described in ref. 4 including MLOGP <4.15 as lipophilicity threshold. The Ghose filter is the method detailed in the original publication^58^, where the atomic log *P* is calculated with WLOGP. The very simple yet efficient Veber filter is implemented directly from the seminal paper^59^. The Egan filter is yielded from the Egan Egg^60^,^75^, but the closed-source ALOGP98 was replaced by WLOGP^17^. The Muegge filter^61^ was adapted to fit SwissADME implementation and usage by calculating XLOGP3 as lipophilicity descriptor. The Bioavailability Score was implemented without changes from Martin *et al*.^62^. Similarly, the leadlikeness filter included in the *Medicinal Chemistry* section was adapted from the original rule^64^ by using XLOGP3 as lipophilicity descriptor.

Both methods for identification of problematic fragments within the *Medicinal Chemistry* section, i.e. PAINS^6^ and Brenk’s Structural Alert^5^, were implemented using the SMARTS recognition capability of OpenBabel API. The SMARTS definitions for PAINS were retrieved from the Filter-it distribution (version 1.0.2, 2013, http://silicos-it.be.s3-website-eu-west-1.amazonaws.com/software/filter-it/1.0.2/filter-it.html). Little formatting and cleansing were needed to obtain a screenable collection of 481 fragments. Brenk provided directly the SMARTS descriptions of 105 unwanted chemical groups in the supplementary material of the seminal paper^5^.

SwissADME Synthetic Accessibility (SA) Score is based primarily on the assumption that the frequency of molecular fragments in ‘really’ obtainable molecules correlates with the ease of synthesis. The fragmental contribution to SA should be favourable for frequent chemical moieties and unfavourable for rare moieties. We examined the ‘All Now’ subset of ZINC database^76^ (version 12, 2014, http://zinc.docking.org/subsets/all-now accessed April 2015) including 12′782′590 compounds immediately deliverable by vendors. This collection was not submitted to any other filter. FP2 fingerprints of all molecules were computed by OpenBabel^9^ thus generating 12′782′590 bit strings (i.e. the fingerprints). The frequency of occupancy of each of the 1024 bits for the entire library was calculated and its contribution to SA obtained by applying natural logarithm to the normalized bit count. As a result, this fragmental system of bits allows extremely fast evaluation of any input molecule by reading its FP2 string. Fragmental contribution of bit occupancy is summed and linearly modulated by corrective factors, which are penalties for size (MW) and complexity. This latter is based on SMARTS recognition of chiral centres, spiro functions, bridged rings and macrocycles (more than 8-membered rings). The coefficients of the corrective terms are those defined by Ertl & Schuffenhauer^11^. Finally the score is normalized to range from 1 (very easy) to 10 (very difficult to synthetize). As crude as it may seem, SwissADME SA Score is extremely fast and performing slightly better than two similar methods previously published^11^,^65^ (refer to Table 2).

### Graphical output

The graphical output of SwissADME consists of the BOILED-Egg directly implemented from ref. 17 to predict passive diffusion through HIA and BBB by position in a WLOGP-*versus*-TPSA physicochemical space. The HIA model was trained on 660 well-absorbed and non-absorbed molecules and a 10-fold crossvalidation returned an accuracy (ACC_CV_) of 92% and a Matthews correlation coefficient (MCC_CV_) of 0.65. The BBB model was built on a training set of 260 permeant and non-permeant molecules and displayed excellent internal statistics as well: ACC_CV_ = 88% and MCC_CV_ = 0.75. The plot is enriched by color-coding referring to P-gp active efflux predicted by a SVM classifier (refer to *Pharmacokinetics*). We aimed at providing a global and efficient analysis of absorption and brain access for multiple molecules at once. For more advanced interactive features, the graph is generated in JavaScript using the Flot plotting library (version 0.8, 2015, http://www.flotcharts.org/). The user has the possibility to visualize the position of the molecules between the different BOILED-Egg compartments and their propensity of being substrate of P-gp by coloured points: blue for substrate (PGP+) and red for non-substrate (PGP−). The dots are also the mean to visualize name and chemical structure of the molecules (generated on-the-fly by the dedicated Chemaxon web server as described above) by leaving the mouse over the dot. As well, it is possible to access the corresponding output panel summarizing all calculated values for the molecule by clicking on the corresponding dot. Getting back to the BOILED-Egg is as simple as clicking on the up arrow at the top-right corner of the panel. All these functionalities, as well as interoperability with other SwissDrugDesign web tools are coded in PHP and Javascript.

## Additional Information

**How to cite this article**: Daina, A. *et al*. SwissADME: a free web tool to evaluate pharmacokinetics, drug-likeness and medicinal chemistry friendliness of small molecules. *Sci. Rep.* **7**, 42717; doi: 10.1038/srep42717 (2017).

**Publisher's note:** Springer Nature remains neutral with regard to jurisdictional claims in published maps and institutional affiliations.

## Supplementary Material

Supplementary Information

## Figures and Tables

**Table 1:** Statistical performance of SVM classification models for substrate or inhibitor of pharmacokinetics-relevant protein, P-gp and CYP.

Model	SwissADME					Previous models					
	TR/TS^[a]^	ACC_CV_^[b]^	AUC_CV_^[c]^	ACC_ext_^[d]^	AUC_ext_^[e]^	TR/TS	ACC_CV_	AUC_CV_	ACC_ext_	AUC_ext_	Reference
P-gp substrate	1033/415	0.72	0.77	0.89	0.94	544/n.c.	0.71^[f]^	n.c.	n.c.	n.c.	53
						484/300	0.64	n.c.	0.59	n.c.	71
						332/n.c.	0.74^[f]^	0.77^[f]^	n.c.	n.c.	15
						212/120	0.74	n.c.	0.88	n.c.	57
CYP1A2 inhibitor	9145/3000	0.83	0.90	0.84	0.91	9145/3000	n.c.	n.c.	0.88	0.95	54
						12099/2804	0.82^[f]^	n.c.	0.68	0.81	77
						7208/7128	0.88^[g]^	n.c.	n.c.	0.93	55
CYP2C19 inhibitor	9272/3000	0.80	0.86	0.80	0.87	9272/3000	n.c.	n.c.	0.85	0.91	54
						11885/2691	0.79^[f]^	n.c.	0.81	0.84	77
						6038/5923	0.81^[g]^	n.c.	n.c.	0.89	55
CYP2C9 inhibitor	5940/2075	0.78	0.85	0.71	0.81	8720/3000	n.c.	n.c.	0.83	0.90	54
						12130/2579	0.78^[f]^	n.c	0.89	0.86	77
						6627/6530	0.83^[g]^	n.c.	n.c.	0.89	55
CYP2D6 inhibitor	3664/1068	0.79	0.85	0.81	0.87	9726/3000	n.c.	n.c.	0.84	0.88	54
						11881/2860	0.84^[f]^	n.c.	0.88	0.88	77
						7788/7761	0.90^[g]^	n.c.	n.c.	0.85	55
CYP3A4 inhibitor	7518/2579	0.77	0.85	0.78	0.86	8893/5135	n.c.	n.c.	0.84	0.92	54
						11536/7025	0.78^[f]^	n.c.	0.76	0.78	77
						2334/6738	0.81^[g]^	n.c.	n.c.	0.87	55

^a^Number of molecules in the training set (TR) and in the test set (TS); ^b^10-fold cross-validation accuracy; ^c^10-fold cross-validation area under receiver operating characteristic (ROC) curve; ^d^external validation accuracy; ^e^external validation area under ROC curve; ^f^5-fold cross-validation; ^g^7-fold cross-validation.

**Table 2:** Statistical performance of synthetic accessibility (SA) scores on two external test sets.

Test set			SwissADME			Original models published with the test set			
	#molecule^[a]^	#chemist^[b]^	MAE^[c]^	RMSE ^[d]^	r^[e]^	MAE	RMSE	r	Reference
1	40	9	0.63	0.81	0.96	0.91	1.13	0.94	11
2	114	4	1.02	1.33	0.62	1.07	1.41	0.62	65

^a^Number of external molecules; ^b^number of chemist marks; ^c^mean average error; ^d^root mean square error; ^e^linear correlation coefficient.
