# Supplementary material for: SwissADME: a free web tool to evaluate pharmacokinetics, drug-likeness and medicinal chemistry friendliness of small molecules
Source: Sci Rep. 2017 Mar 3;7:42717. doi: 10.1038/srep42717 (PMC5335600; doi:10.1038/srep42717)
Supplement: Supplementary Information [file srep42717-s1.pdf]

Supplementary Information to:

SwissADME: a free web tool to evaluate pharmacokinetics, drug-likeness and medicinal chemistry friendliness of small molecules

Antoine Daina[a], Olivier Michielin*[,a,b,c] and Vincent Zoete*[,a]

[a]SIB Swiss Institute of Bioinformatics, Molecular Modeling Group, Quartier Sorge, Bâtiment Génopode, CH-1015 Lausanne, Switzerland
[b]Department of Oncology, Centre Hospitalier Universitaire Vaudois, CH-1015 Lausanne, Switzerland
[c]Ludwig Institute for Cancer Research, University of Lausanne, CH-1015 Lausanne, Switzerland

*Coresponding authors:
E-mail: vincent.zoete@sib.swiss
E-mail: olivier.michielin@sib.swiss

Table S1. Pool of molecular and physicochemical descriptors evaluated for building SVM models.

#	variable	description	ref
**1**	nF	number of fluorine atoms	1
**2**	sbonds	number of single bonds	1
**3**	dbonds	number of double bond	1
**4**	tbonds	number of triple bond	1
**5**	abonds	number of aromatic bond	1
**6**	GetMolWt	molecular weight	1
**7**	NumAtoms	number of atoms	1
**8**	NumHvyAtoms	number of heavy atoms	1
**9**	AP	aromatic portion	2
**10**	NumAcceptor	number of H-bond acceptors	1
**11**	NumDonor	number of H-bond donors	1
**12**	NumCarbon	number of carbon atoms	1
**13**	NumHetero	number of heteroatoms	1
**14**	NumAromatic	number of aromatic atoms	1
**15**	NumRotors	number of rotatable bonds	1
**16**	NumRing	number of rings	1
**17**	TPSA	topological surface area	3
**18**	MR	molecular refractivity	1
**19**	MlogpCX	weighted sum for carbon and halogen atoms	4
**20**	MlogpNO	total number of nitrogen and oxygen atoms	4
**21**	MlogpUB	number of unsaturated bonds	4
**22**	MlogpRNG	presence of ring structures	4
**23**	MlogpNO2	number of nitro groups	4
**24**	MlogpNCS	presence of thiocyanate or isothiocynanate	4
**25**	MlogpQN	prensence of quaternary nitrogen or N-oxide	4
**26**	MlogpALK	presence of alkane, alkene cyclocalkane or cycloalkene	4
**27**	MlogpHB	presence of intramolecular H-bond	4
**28**	MlogpPOL	number of aromatic substituents	4
**29**	MlogpBLM	presence of beta-lactam	4
**30**	MlogpAMP	presence of amphoteric property	4
**31**	MlogpPRX	proximitry effect of nitrogen and oxygen atoms	4
**32**	mlogp	MLOGP	4
**33**	mhlogp	NC+NHET log *P*	5
**34**	alogp	WLOGP	6
**35**	logP	OpenBabel log *P*	1
**36**	NumSpiro	number of spiro groups	7
**37**	NumBridge	number of ringbridging atoms	7
**38**	NumStereo	number of stereocenters	7
**39**	NumMacrocycle	number of macrocycles	7
**40**	sizePenalty	size penalty	7
**41**	macrocyclePenalty	macrocycle penalty	7
**42**	stereoComplexity	stereo complexity	7
**43**	ringComplixity	ring complexity	7
**44**	complexityPenalty	complexity penalty	7
**45**	ilogp	ILOGP	8
**46**	xlogp3	XLOGP3	9
**47**	silicos_logP	Filter-IT log *P*	*a*
**48**	logSwE	ESOL log *S*	2
**49**	logSwA	Ali log *S*	10
**50**	silicos_logS	Filter-IT log *S*	*a*

[a] FILTER-IT (version 1.0.2) 2013, http://silicos-it.be.s3-website-eu-west-1.amazonaws.com/software/filter-it/1.0.2/filter-it.html

Table S2. Selection and spread of the 16 molecular and physicochemical descriptors for Pgp-subtrate SVM model

descriptors[a]	minimum value in TR[b]	maximum value in TR[c]
**dbonds (3)**	0	19
**NumAtoms (7)**	9	279
**NumDonor (11)**	0	21
**NumRotors (15)**	0	69
**NumRing (16)**	0	13
**MlogpNO (20)**	0	37
**MlogpUB (21)**	0	36
**MlogpRNG (22)**	0	1
**mhlogp (33)**	-0.41	8.39
**logP (35)**	-4.56	18.48
**NumStereo (38)**	0	25
**ilogp (45)**	-8.9	12.57
**xlogp3(46)**	-5.82	19.06
**silicos_logP(47)**	-15.61	19.51
**logSwE(48)**	-15.57	2.1
**silicos_logS(50)**	-23.02	5.97

[a] name of variable and number in brackets, refer to Supplementary Table S1. Descriptors were selected for significance (F-score), broadness (number of non-zero values and coefficient of variation) and orthogonality (correlation coefficient)

[b] miniumum values for each descriptors among the 1033 molecules of the training set (TR)

[c] maximum values for each descriptors among the 1033 molecules of the training set (TR)

Table S3. Selection and spread of the 20 molecular and physicochemical descriptors for CYP1A2 inhibitor SVM model

descriptors[a]	minimum value in TR[b]	maximum value in TR[c]
**sbond (2)**	4	184
**dbonds (3)**	0	19
**abonds (5)**	0	48
**NumHvyAtoms (8)**	2	114
**AP (9)**	0	1
**NumAcceptor (10)**	0	26
**NumDonor (11)**	0	19
**NumRotors (15)**	0	37
**NumRing (16)**	0	13
**MlogpNO (20)**	0	37
**MlogpRNG (22)**	0	1
**MlogpPOL (28)**	0	14
**MlogpPRX (31)**	0	31
**mlogp (32)**	-8.83	10.17
**alogp (34)**	-11.55	20.75
**stereoComplexity (42)**	0	1.45
**ilogp (45)**	-15.05	8.83
**xlogp3(46)**	-8.89	25.90
**silicos_logP (47)**	-8.90	21.57
**silicos_logS (50)**	-25.81	6.01

[a] name of variable and number in brackets, refer to Supplementary Table S1. Descriptors were selected for significance (F-score), broadness (number of non-zero values and coefficient of variation) and orthogonality (correlation coefficient)

[b] miniumum values for each descriptors among the 9145 molecules of the training set (TR)

[c] maximum values for each descriptors among the 9145 molecules of the training set (TR)

Table S4. Selection and spread of the 21 molecular and physicochemical descriptors for CYP2C19 inhibitor SVM model

descriptors[a]	minimum value in TR[b]	maximum value in TR[c]
**dbonds (3)**	0	17
**GetMolWt (6)**	33.03	1550.18
**AP (9)**	0	1
**NumAcceptor (10)**	0	26
**NumDonor (11)**	0	19
**NumAromatic (14)**	0	56
**NumRotors (15)**	0	37
**NumRing (16)**	0	15
**TPSA (17)**	0	551.01
**MlogpNO (20)**	0	33
**MlogpPOL (28)**	0	14
**MlogpPRX (31)**	0	31
**mlogp (32)**	-7.44	10.17
**mhlogp (33)**	0.47	10.26
**logP (35)**	-5.67	20.75
**stereoComplexity (42)**	0	1.45
**complexityPenalty (44)**	0.04	7.39
**ilogp (45)**	-15.71	8.83
**xlogp3(46)**	-8.89	25.90
**silicos_logP (47)**	-8.90	21.57
**silicos_logS (50)**	-25.81	6.01

[a] name of variable and number in brackets, refer to Supplementary Table S1. Descriptors were selected for significance (F-score), broadness (number of non-zero values and coefficient of variation) and orthogonality (correlation coefficient)

[b] miniumum values for each descriptors among the 9272 molecules of the training set (TR)

[c] maximum values for each descriptors among the 9272 molecules of the training set (TR)

Table S5. Selection and spread of the 23 molecular and physicochemical descriptors for CYP2C9 inhibitor SVM model

descriptors[a]	minimum value in TR[b]	maximum value in TR[c]
**dbonds (3)**	0	17
**GetMolWt (6)**	33.03	1449.25
**AP (9)**	0	1
**NumAcceptor (10)**	0	26
**NumDonor (11)**	0	19
**NumCarbon (12)**	0	88
**NumRotors (15)**	0	37
**NumRing (16)**	0	10
**TPSA (17)**	0	556.17
**MlogpUB (21)**	0	40
**MlogpRNG (22)**	0	1
**MlogpNO (23)**	0	2
**MlogpPRX (31)**	0	31
**mlogp (32)**	-7.44	10.17
**mhlogp (33)**	0.47	10.26
**logP (35)**	-5.67	20.75
**NumSpiro (36)**	0	3
**sizePenalty (40)**	0.04	5.63
**stereoComplexity (42)**	0	1.32
**ilogp (45)**	-15.06	8.83
**silicos_logP (47)**	-7.04	21.57
**logSwA (49)**	-29.83	5.18
**silicos_logS (50)**	-25.81	4.67

[a] name of variable and number in brackets, refer to Supplementary Table S1. Descriptors were selected for significance (F-score), broadness (number of non-zero values and coefficient of variation) and orthogonality (correlation coefficient)

[b] miniumum values for each descriptors among the 5940 molecules of the training set (TR)

[c] maximum values for each descriptors among the 5940 molecules of the training set (TR)

Table S6. Selection and spread of the 17 molecular and physicochemical descriptors for CYP2D6 inhibitor SVM model

descriptors[a]	minimum value in TR[b]	maximum value in TR[c]
**sbond (2)**	3	154
**dbonds (3)**	0	17
**AP (9)**	0	1
**NumDonor (11)**	0	15
**NumHetero (13)**	0	35
**NumAromatic (14)**	0	56
**NumRotors (15)**	0	37
**NumRing (16)**	0	15
**TPSA (17)**	0	551.01
**MlogpCX (19)**	0	62
**MlogpPRX (31)**	0	31
**mlogp (32)**	-7.44	8.53
**logP (35)**	-4.29	13.34
**ilogp (45)**	-15.71	8.17
**xlogp3(46)**	-8.53	13.24
**silicos_logP (47)**	-6.46	15.22
**silicos_logS (50)**	-23.68	3.43

[a] name of variable and number in brackets, refer to Supplementary Table S1. Descriptors were selected for significance (F-score), spread (number of non-zero values and coefficient of variation) and orthogonality (correlation coefficient)

[b] miniumum values for each descriptors among the 3664 molecules of the training set (TR)

[c] maximum values for each descriptors among the 3664 molecules of the training set (TR)

Table S7. Selection and spread of the 21 molecular and physicochemical descriptors for CYP3A4 inhibitor SVM model

descriptors[a]	minimum value in TR[b]	maximum value in TR[c]
**sbond (2)**	3	184
**dbonds (3)**	0	19
**abonds (5)**	0	60
**AP (9)**	0	1
**NumAcceptor (10)**	0	38
**NumDonor (11)**	0	21
**NumHetero (13)**	0	42
**NumRotors (15)**	0	35
**NumRing (16)**	0	15
**MlogpCX (19)**	0	72
**MlogpRNG (22)**	0	1
**MlogpPOL (28)**	0	14
**MlogpPRX (31)**	0	31
**mlogp (32)**	-13.72	9.87
**logP (35)**	-14.02	13.34
**NumStereo (38)**	0	38
**stereoComplexity (42)**	0	1.59
**ilogp (45)**	-16.31	7.93
**silicos_logP (47)**	-13.31	13.64
**logSwE(48)**	-13.93	3.82
**silicos_logS (50)**	-23.68	11.15

[a] name of variable and number in brackets, refer to Supplementary Table S1. Descriptors were selected for significance (F-score), broadness (number of non-zero values and coefficient of variation) and orthogonality (correlation coefficient)

[b] miniumum values for each descriptors among the 7518 molecules of the training set (TR)

[c] maximum values for each descriptors among the 7518 molecules of the training set (TR)

Table S8. Predictive capability of SVM models on external test sets

model	TS+[a]	TS-[b]	ACC[c]	TPR[d]	SPC[e]	AUC[f]
**P-gp substrate**	215	200	0.89	0.89	0.89	0.94
**CYP1A2 inhibitor**	1412	1588	0.84	0.84	0.84	0.91
**CYP2C19 inhibitor**	1386	1614	0.80	0.80	0.80	0.87
**CYP2C9 inhibitor**	1020	1055	0.71	0.73	0.70	0.81
**CYP2D6 inhibitor**	528	540	0.81	0.80	0.81	0.87
**CYP3A4 inhibitor**	1289	1290	0.78	0.79	0.77	0.86

[a] number of positive molecules in the test set (TS)

[b] number of negative molecules in the test set (TS)

[c] accuracy

[d] sensitivity

[e] specificity

[f] area under ROC curve

Supplementary References

1. O'Boyle, N. M. *et al*. OpenBabel: An open chemical toolbox. *J. Cheminform*. **3,** 33 (2011).
2. Delaney, J. S. ESOL: Estimating Aqueous Solubility Directly from Molecular Structure. *J Chem Inf Model* **44,** 1000–1005 (2004).
3. Ertl, P., Rohde, B. & Selzer, P. Fast Calculation of Molecular Polar Surface Area as a Sum of Fragment-Based Contributions and Its Application to the Prediction of Drug Transport Properties. *J. Med. Chem*. **43,** 3714–3717 (2000).
4. Moriguchi, I., Shuichi, H., Nakagome, I. & Hirano, H. Comparison of reliability of log P values for Drugs calculated by several methods. *Chem Pharm Bull* **42,** 976–978 (1994).
5. Mannhold, R., Poda, G. I. & Ostermann, C. Calculation of molecular lipophilicity: State-of-the-art and comparison of log P methods on more than 96,000 compounds. *J Pharm Sci* **98,** 861–893 (2009).
6. Wildman, S. A. & Crippen, G. M. Prediction of Physicochemical Parameters by Atomic Contributions. *J Chem Inf Model* **39,** 868–873 (1999).
7. Ertl, P. & Schuffenhauer, A. Estimation of synthetic accessibility score of drug-like molecules based on molecular complexity and fragment contributions. *J. Cheminform*. **1,** 8 (2009).
8. Daina, A., Michielin, O. & Zoete, V. iLOGP: A Simple, Robust, and Efficient Description of n-Octanol/Water Partition Coefficient for Drug Design Using the GB/SA Approach. *J Chem Inf Model* **54,** 3284–3301 (2014).
9. Wang, R., Fu, Y. & Lai, L. A New Atom-Additive Method for Calculating Partition Coefficients. *J Chem Inf Model* **37,** 615–621 (1997).
10. Ali, J., Camilleri, P., Brown, M. B., Hutt, A. J. & Kirton, S. B. Revisiting the general solubility equation: in silico prediction of aqueous solubility incorporating the effect of topographical polar surface area. *J Chem Inf Model* **52,** 420–428 (2012).
